# Case Study of a Broken Instrument in a Primary Tooth and Literature Review

**DOI:** 10.3390/children12020149

**Published:** 2025-01-27

**Authors:** Masashi Nakano, Tatsuya Akitomo, Masashi Ogawa, Mariko Kametani, Momoko Usuda, Satoru Kusaka, Chieko Mitsuhata, Ryota Nomura

**Affiliations:** 1 Department of Pediatric Dentistry, Graduate School of Biomedical and Health Sciences, Hiroshima University, 1-2-3 Kasumi, Minami-ku, Hiroshima 734-8553, Japan; nakano@saitamada.or.jp (M.N.); caries0@hiroshima-u.ac.jp (M.O.); mrysk25@hiroshima-u.ac.jp (M.K.); musuda@hiroshima-u.ac.jp (M.U.); chiekom@hiroshima-u.ac.jp (C.M.); rnomura@hiroshima-u.ac.jp (R.N.); 2 Oral Health Center of Saitama Dental Association, Saitama 330-0075, Japan; 3 Department of Pediatric Dentistry, Hiroshima University Hospital, Hiroshima 734-8551, Japan; higechi@hiroshima-u.ac.jp

**Keywords:** broken instrument, primary tooth, literature review

## Abstract

Background: Root canal treatment is an important element of dental treatment, and broken instruments are one cause of endodontic treatment failure. Broken instruments are rarely reported in primary teeth because of their inherently wider and relatively straighter root canals. We describe a case of a patient with a broken instrument in a primary tooth and reviewed the literature across three databases. Case presentation: A boy aged 7 years and 2 months was referred to our hospital because of pain persisting despite multiple dental treatments. Radiographic examination revealed a broken instrument in the maxillary right primary second molar. The patient experienced dental fear, so the treatment proceeded with the use of behavioral management and nitrous oxide, after which his pain disappeared. At the age of 9 years and 2 months, eruption of the successive permanent teeth was confirmed, and no pathological findings were observed except enamel hypoplasia. We undertook a literature review across three databases and found only two articles about broken instruments in primary teeth, neither of which described the eruption of the permanent teeth. All three cases involved primary molars. Conclusions: We removed a broken instrument from the root of a primary molar and performed root canal treatment, resulting in a good outcome for the primary molar until it was replaced with successive permanent teeth. Although rare, broken instruments in the roots of primary molars do occur, and care should be taken during root canal treatment of primary molars.

## 1. Introduction

Dental caries and periodontitis are the most common oral diseases caused by oral microbiota [1,2,3]. Recently, the prevalence of dental caries in many developed countries, including Japan, has decreased [4,5,6]. Additionally, oral health status in these countries has become more polarized and patients still present with severe caries [4,5,6]. Tertiary teaching institutions such as university hospitals treat patients with a variety of conditions, including dental abnormalities and dental trauma, as well as hospitalized patients [7,8,9,10,11,12]. However, dental caries remains the most common complaint among patients visiting the dental hospital in tertiary teaching institutions in Japan [8,13,14].

Nonsurgical root canal treatment is an important element of comprehensive dental healthcare [15,16,17]. Previous studies have reported success rates of >90% for nonsurgical root canal treatment [17,18,19]. However, the absence of an apical seal (i.e., apical leakage) is the most common cause of endodontic treatment failure [20]. Broken instruments result from procedural errors during root canal treatment, and despite the attempts of manufacturers to improve the design of the instruments, fracture remains a problem in endodontic treatment [17,20,21].

Although there are some case reports of fractured root canal instruments in permanent teeth, there have been few reports involving primary teeth [22,23,24]. We encountered a patient with a broken instrument in a primary tooth. We present the case report and a literature review regarding broken instruments in primary teeth.

## 2. Detailed Case Description

A boy aged 7 years and 2 months was referred to our hospital for detailed examination of the maxillary right primary molars. He had visited a private dental clinic complaining of pain in the maxillary right primary molars 5 months previously and received root canal treatment. However, he experienced pain when the temporary restoration was placed. He was taken to another private dental clinic 1 month later, but his pain always occurred after a dental procedure. He developed a fever, which led to visiting the emergency room where they removed the temporary restoration. Additionally, he became an uncooperative patient during dental treatment, resulting in a referral to our hospital. There was no relevant medical or family history.

**Figure 1:**
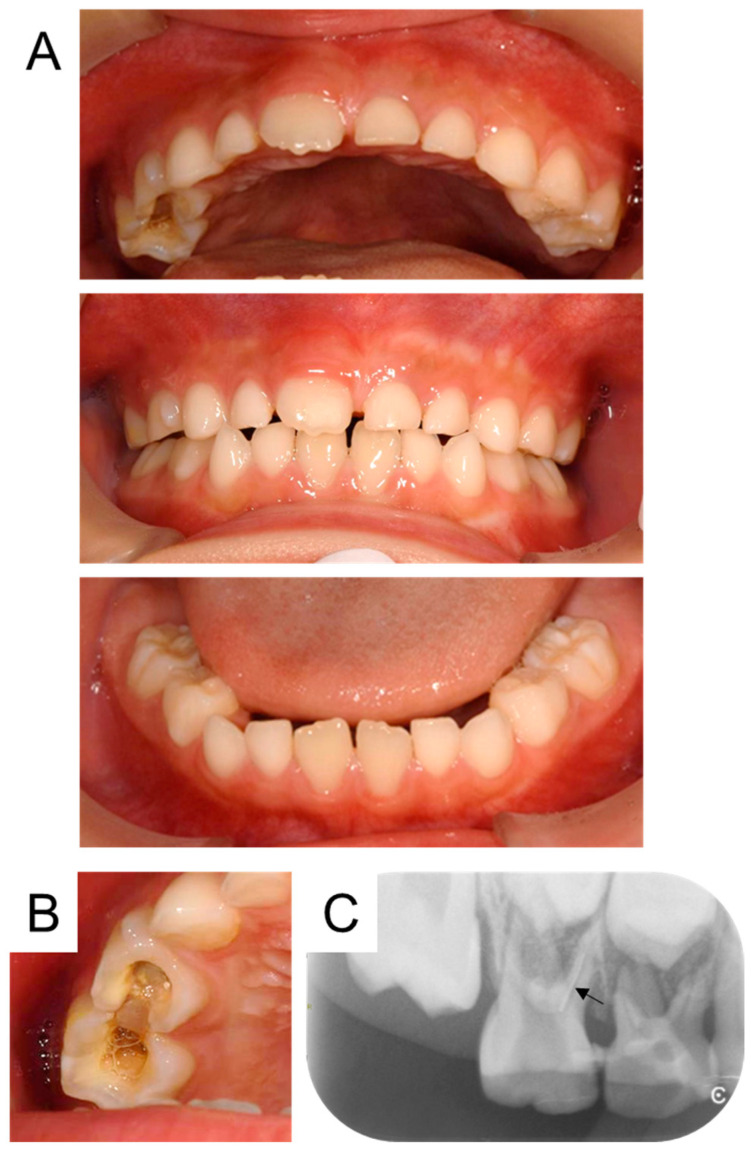
First examination at age 7 years and 2 months. Intraoral photographs (**A**,**B**). Radiographic examination showing a suspected broken instrument (arrow) in the mesio-buccal canal of the maxillary right second primary molar (**C**).

At the first visit, intraoral photographs showed three erupted permanent central incisors and 17 primary teeth in the oral cavity (Figure 1A). The maxillary right primary molars were connected with composite resin, and there was no temporary restoration (Figure 1B). The discharge of pus from the gum was detected. Radiographic examination revealed bone resorption in the region of the maxillary right primary molars and a radiopacity was observed in the mesio-buccal canal of the second primary molar, suggesting the presence of a broken instrument (Figure 1C). Dental caries were also present in other primary molars.

**Figure 2:**
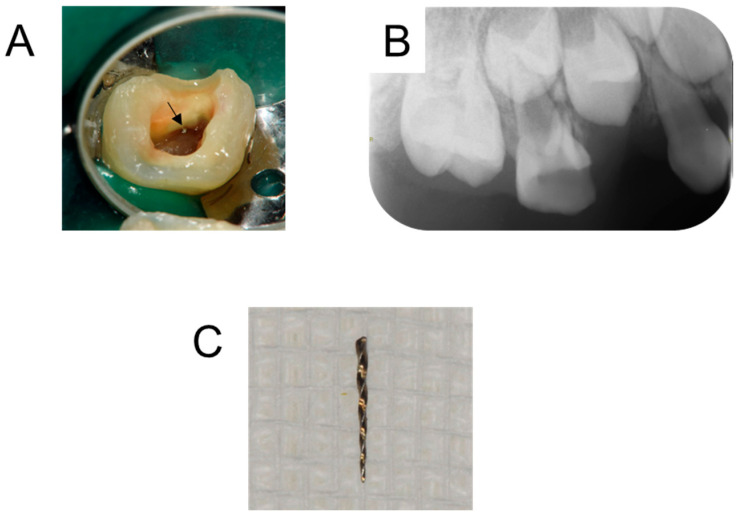
Images at age 7 years and 4 months. Intraoral photograph during dental treatment showing broken instrument (arrow) (**A**). Radiograph confirming removal of the instrument (**B**). Photograph of the removed broken instrument (**C**).

**Figure 3:**
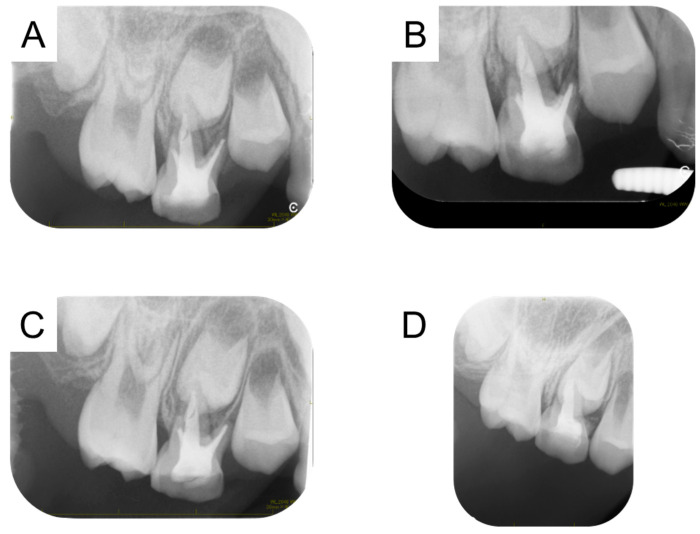
Radiographs of the maxillary right primary second molar: 7 years and 7 months (**A**), 7 years and 10 months (**B**), 8 years (**C**), and 8 years and 10 months (**D**).

We diagnosed apical periodontitis of the maxillary right primary molars, and a treatment plan was formulated. Due to dental fear, behavioral management was performed before the start of dental treatment. In brief, the patients touch and operate the dental instruments, which leads to a reduction in dental fear. In addition, the dental treatment was performed under nitrous oxide inhalation sedation to reduce the patient’s fear during dental treatment. The maxillary right primary first molar was unable to be saved; it was extracted after the inflammation was controlled. The broken instrument located in the mesio-buccal canal of the maxillary right primary second molar was removed using ultrasonic tips (Figure 2A). Radiographic examination confirmed that the instrument had been removed from the root canal (Figure 2B). The removed broken instrument was a reamer approximately 5 mm in length (Figure 2C). The root canal treatment was completed 1 month later, and the tooth was restored with glass-ionomer cement. Two months later, there was no pain, and no pathological findings could be seen on the periapical radiograph (Figure 3A). During the follow-up period, inflammatory absorption of the mesio-buccal canal was found at age 8 years and 10 months, and the tooth was extracted 2 months later (Figure 3B–D).

**Figure 4:**
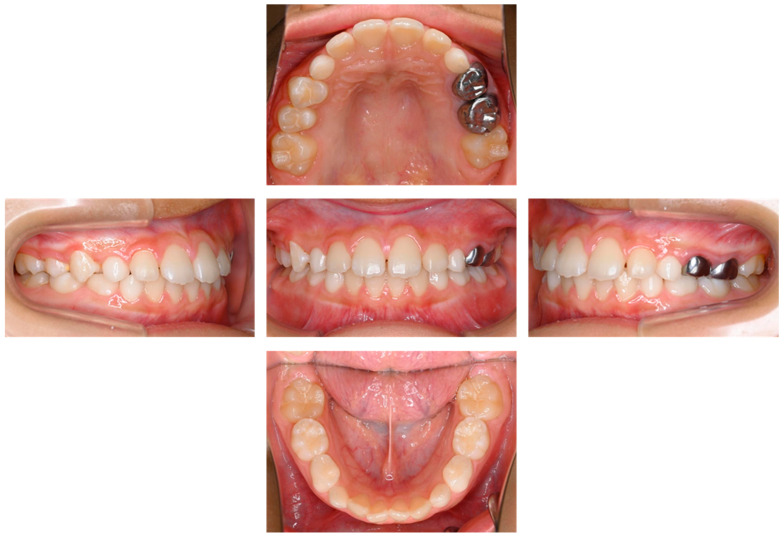
Intraoral photographs at age 10 years.

**Figure 5:**
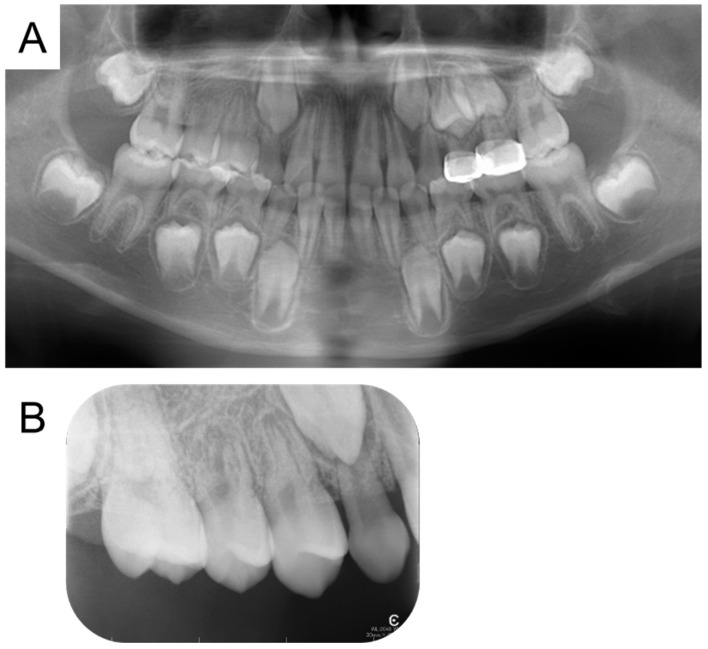
Radiographs at age 10 years: panoramic photograph (**A**), and periapical photograph (**B**).

The maxillary right second premolar erupted at the age of 9 years and 2 months. Enamel hypoplasia was detected on the mesial surface, but no symptoms were reported. There were no pathological findings in the intraoral examination or radiographic examination at 10 years of age (Figure 4 and Figure 5).

## 3. Methods

### 3.1. Search Strategy

The review protocol was developed in accordance with the Preferred Reporting Items for Systematic Reviews and Meta-Analyses (PRISMA) statement and previous reports [25,26]. A literature review of three databases (Web of Science, Scopus, and PubMed) was conducted by one of the authors on 6 December 2024. The articles were searched for manually using the terms “broken instrument” and “primary tooth”. 

### 3.2. Inclusion Criteria

The inclusion criteria for this study included the following:

- Articles that could be viewed in their entirety.
- Articles with their full text in English.
- Case reports about broken instruments in primary teeth.

### 3.3. Exclusion Criteria

The exclusion criteria for this study included the following:

- Articles that were not suitable for the objective of this review.
- Articles that used the wrong study design.
- Not being a case report, such as review.

### 3.4. Study Selection

According to the inclusion and exclusion criteria, one dentist performed the literature analysis and selected articles for this review.

### 3.5. Data Extraction

Data were extracted from the articles about tooth number, root canal, type of instrument, X-ray follow-up, and influence on successive teeth.

## 4. Results

Our literature search uncovered 25 articles in three databases (Web of Science: 9, Scopus: 7, PubMed: 9). Duplicates were removed and 16 articles remained. Of the 16 articles, two fulfilled the selection criteria. Detailed information about the articles is shown in Table 1 [27,28]. Thus, including the present case, we reviewed a total of three cases of broken instruments in primary teeth.

All three cases occurred in a primary molar, and few cases were reported in primary incisors. All cases were on the right side, with no predilection for maxilla over mandible, nor for particular root canals. Various types of instruments were involved. Both previous cases were followed up with X-ray examinations; however, no information was provided about the successive permanent teeth.

## 5. Discussion

It has been reported that the prevalence of fracture of stainless-steel hand files is 2–6%, while that of NiTi rotary files is 1.04–13.54% [29,30,31,32,33,34]. However, the separation of a file during pulpectomy rarely occurs in primary teeth because of the inherently wider and relatively straighter root canals [27]. Additionally, primary teeth eventually fall out, so there are few cases of broken instruments that require treatment. We encountered a patient with a broken instrument in a primary tooth. We also undertook a literature review. To our knowledge, this is the first report of a patient with a broken instrument in a primary tooth who was followed up until permanent tooth eruption.

Instrument fracture during root canal therapy is a troublesome incident that can interfere with efficient cleaning and shaping of the root canal and can act as an irritant to the periapical tissues, especially when the separated fragment overextends from the root apex [35,36,37,38]. In the present case, multiple root canal treatments in private dental clinics could not resolve the patient’s symptoms. As a consequence, the patient gradually developed dental phobia and was unable to cooperate with dental treatment, resulting in a referral to our hospital. At his first visit, the patient reported feeling pain, and discharge of pus from the gum was detected. Negative dental experiences, especially those resulting from dental pain, can lead to the development of fear and anxiety, which in turn can lead to the avoidance of further dental treatment [39,40,41,42]. The incidence of pediatric dental fear is higher than that of adult dental fear and, if left unchecked, can persist for a lifetime and adversely impact the patient’s physical and psychological health [43,44,45]. We chose behavioral management administrated before dental treatment, which led to a reduction in his dental fear. In addition, inhalation sedation with nitrous oxide is a form of light conscious sedation widely used in apprehensive children to help them relax and accept dental treatment [46]. Our patient was able to receive dental treatment without physical restraint by using behavioral management and nitrous oxide. Appropriate treatment and management improved not only the patient’s dental condition, but also his dental fear.

A literature search was conducted for three databases, and only two previous reports were located. The prevalence of dental caries has been decreasing in developed countries [4,5,6]. As mentioned above, the anatomical characteristic of primary teeth makes it unlikely for broken instrument [27]. In addition, broken instruments are rarely an issue because primary teeth eventually fall out. This background may be why so few articles met the criteria. We reviewed three cases including the present case, and all occurred in primary molars. Terauchi et al. (2022) reported that instruments are fractured more commonly in molars than in anterior teeth because of factors including canal accessibility, root canal diameter, and root canal curvature [47]. The same tendency may also pertain to primary teeth. Pedir et al. (2016) reported that 94% of fractured instruments occurred in molars, with 66% in the mesio-buccal canal, followed by 24% in the mesio-lingual canal [48]. In two out of the three cases, the instrument was broken in the root canal of a primary molar, suggesting that these root canals are at high risk of instrument fracture in primary teeth. Reciprocating file systems have a lower incidence of instrument fracture than rotary file systems [47,49,50,51]. However, the types of instruments used in these three cases were different. Although the sample size was small, factors such as the operating method and metal fatigue of the instruments may be more important than the type of instrument. Future investigations including other case reports may lead to the identification of factors influencing broken instruments in primary teeth.

Premature loss of the primary teeth may lead to a lack of space, malocclusion, and midline discrepancies in the permanent dentition [52,53,54]. Additionally, premature loss of primary molars impacts the oral health-related quality of life of children [55]. At the patient’s first visit, the first molar had not erupted, and we decided to preserve the primary molars until space maintenance was possible. The technique of using ultrasonic tips under a dental operating microscope is considered to be the optimal strategy for the successful removal of broken instruments [56,57,58,59]. In the two previous cases, the instruments were also removed in this way [27,28]. In the present case, the broken instrument was clearly visible, permitting removal using only ultrasonic tips. These cases demonstrate the usefulness of ultrasonic tips for the removal of broken instruments in primary teeth.

At the age of 8 years and 10 months, radiographic examination revealed inflammatory resorption of the mesio-buccal canal, necessitating extraction. Additionally, enamel hypoplasia was detected on the mesial surface of the maxillary right second premolar, which was located in the region of the broken instrument. Dental abnormalities can occur in healthy children; therefore, the effect of the broken instrument on the inflammatory root resorption or enamel hypoplasia is unclear [60,61,62]. However, no other abnormal findings and no malocclusion such as a space deficit were detected. Long-term oral management resulted in a minimal impact on the permanent teeth. Regular dental checkups are important for the early detection of disease, and long-term oral management also leads to improvement in patients’ quality of life [63,64,65]. This report highlights the importance of long-term follow-up. 

This study has some limitations. First, it was unclear when the instrument was broken. Second, the patient felt pain every time a temporary restoration was placed at the private dental clinics, and the association between the pain and the broken instrument was unclear. A retained broken instrument does not have a significant adverse effect on the quality of the root canal seal by filling materials, and the success of endodontic treatment mainly depends on the coronal seal and effective cleaning of the middle and coronal thirds of the root [20,66]. The pain was likely caused by inflammation, because it disappeared after the abscess was incised. Third, the sample size was small even though the literature search spanned three databases. This report brings together previous reports at once reflecting a gap in the existing literature, but the small sample size limits the generalizability of the findings. Breakage of instruments is inevitable during dental procedures. Dental professionals should be aware of such cases, even in primary teeth, where broken instruments are thought to be uncommon. In addition, if such a case is encountered, it is important to follow up and report the outcome until the permanent tooth eruption. Sharing information among dental professionals and increasing sample sizes leads to clarifying the effect of the broken instrument on the root canal of the primary tooth.

## 6. Conclusions

In this case, a broken instrument found in the root canal of a primary molar of a 7-year-old boy was removed using an ultrasonic tip. We then performed root canal treatment on the primary tooth, which saved the tooth and minimized any adverse effects on the successive permanent teeth. Our literature review found that the breakage of instruments in primary teeth is rare, but broken instruments may have adverse effects not only on the primary tooth but also on the successive permanent tooth. Therefore, we should take great care when performing root canal treatment in children.

## Figures and Tables

**Table 1:** Cases of broken instruments in primary teeth reported in the literature.

Author	Tooth	Root Canal	Type of Instrument	X-Ray Follow-Up	Influence on Successive Teeth
Pk M, 2016 [27]	#84	Mesio-lingual canal	H file	15 months	Not listed
Kaul R, 2022 [28]	#85	Disto-buccal canal	K file	6 months	Not listed
Present case	#55	Mesio-buccal canal	Reamer	17 months	Enamel hypoplasia

## Data Availability

Data are contained within the article.
